# Clear cell renal cell carcinoma associated microRNA expression signatures identified by an integrated bioinformatics analysis

**DOI:** 10.1186/1479-5876-11-169

**Published:** 2013-07-10

**Authors:** Jiajia Chen, Daqing Zhang, Wenyu Zhang, Yifei Tang, Wenying Yan, Lingchuan Guo, Bairong Shen

**Affiliations:** 1 Center for Systems Biology, Soochow University, Suzhou 215006, China; 2 School of Chemistry, Biology and Material Engineering, Suzhou University of Science and Technology Suzhou 215009, China; 3 Department of Pathology, School of Medicine, Soochow University, Suzhou 215123, China

**Keywords:** Meta-analysis, Network biomarker, MicroRNA, Clear cell renal cell carcinoma, Pathway analysis, Heterogeneity

## Abstract

**Background:**

Clear cell renal cell carcinoma (ccRCC) represents the most invasive and common adult kidney neoplasm. Mounting evidence suggests that microRNAs (miRNAs) are important regulators of gene expression. But their function in tumourigenesis in this tumour type remains elusive. With the development of high throughput technologies such as microarrays and NGS, aberrant miRNA expression has been widely observed in ccRCC. Systematic and integrative analysis of multiple microRNA expression datasets may reveal potential mechanisms by which microRNAs contribute to ccRCC pathogenesis.

**Methods:**

We collected 5 public microRNA expression datasets in ccRCC versus non-matching normal renal tissues from GEO database and published literatures. We analyzed these data sets with an integrated bioinformatics framework to identify expression signatures. The framework incorporates a novel statistic method for abnormal gene expression detection and an in-house developed predictor to assess the regulatory activity of microRNAs. We then mapped target genes of DE-miRNAs to different databases, such as GO, KEGG, GeneGo etc, for functional enrichment analysis.

**Results:**

Using this framework we identified a consistent panel of eleven deregulated miRNAs shared by five independent datasets that can distinguish normal kidney tissues from ccRCC. After comparison with 3 RNA-seq based microRNA profiling studies, we found that our data correlated well with the results of next generation sequencing. We also discovered 14 novel molecular pathways that are likely to play a role in the tumourigenesis of ccRCC.

**Conclusions:**

The integrative framework described in this paper greatly improves the inter-dataset consistency of microRNA expression signatures. Consensus expression profile should be identified at pathway or network level to address the heterogeneity of cancer. The DE-miRNA signature and novel pathways identified herein could provide potential biomarkers for ccRCC that await further validation.

## Background

Renal cell carcinoma (RCC) represents the leading cause of death among urological malignancies [1]. Clear cell renal cell carcinoma (ccRCC) is the most common histological subtype of RCC. Early stage of renal cancers do not usually cause symptom and it’s difficult to establish an accurate diagnose. ccRCC is relatively resistant to chemotherapy or radiotherapy [2] and the overall clinical outcome is poor [3]. Thus the need for diagnostic and prognostic biomarkers in ccRCC is urgent. Nevertheless, to our knowledge there are still no biomarkers in routine clinical practice in ccRCC.

microRNAs are single-stranded, non-coding RNAs that regulate gene expression at the post-transcriptional level [4]. Aberrant changes in microRNA expression have been shown to be associated with human malignancies [5,6]. Various studies have investigated the miRNA profile in ccRCC lesions in comparison to non-tumoral kidney tissues by microarray technologies [7-13], RT-qPCR [14-16] and more recently with next generation sequencing [17-19]. Altered expression of miRNAs in ccRCC has been frequently reported. Nevertheless, the DE-miRNA lists from different laboratories vary widely due to the inter-platform differences and the limited sample sizes. Potential mechanisms by which miRNAs contribute to ccRCC pathogenesis are still poorly understood.

**Figure 1:**
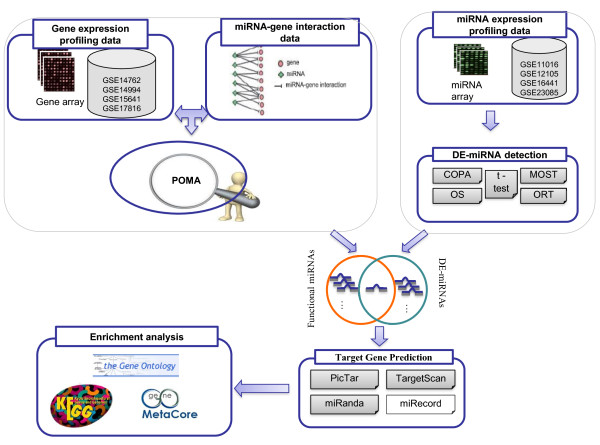
Schematic diagram depicting the analysis pipeline in this study.

Cancer is a systems biology disease, therefore the biomarkers discovery should take into account the heterogeneity and complexity of carcinogenesis. There is a growing movement from individual marker discovery to a systems-oriented paradigm. Due to this recognition, we describe herein an integrated bioinformatics approach to obtain a consistent microRNA expression signature as well as novel microRNA-regulated molecular pathways that contribute to the pathogenesis of ccRCC. The analysis pipeline of this paper is outlined in Figure 1.

## Methods

### Dataset collection

Five publicly available microRNA expression datasets on ccRCC versus non-matching normal renal tissues were downloaded, four of them from the GEO (Gene Expression Omnibus) database, and one from supplementary materials of published literature. Table 1 gives detailed information of the miRNA expression datasets including the original statistical methods used for the DE-miRNA identification. Four mRNA expression datasets by Affymetrix arrays were also extracted from GEO (detailed information given as Additional file 1). All the datasets are downloaded in raw data file format. Probe sequences were mapped to Sanger miRBase 18 [20] for unified miRNA names.

**Table 1:** Summary of microRNA expression datasets used in this study

**GEO accession**	**Reference**	**Platforms**	**Probe Number**	**Number of samples**		**Statistics**
				***normal tissue***	***cancer tissue***	
GSE11016	[6]	LC MRA-1001	835	17	17	t-test
GSE12105	[7]	Agilent Human microRNA Microarray	490	12	12	t-test
GSE16441	[8]	Agilent Human microRNA Microarray	851	8	8	SAM
GSE23085	[10]	LC MRA-1001	881	20	20	t-test
Weng	[18]	Agilent Human microRNA Microarray	723	3	3	t-test

### Data processing

The quantified probing signals were background corrected using Normexp, with offset value set as 0. The background-subtracted data were normalized using the Quantile algorithm. Averages were derived from all quantile normalized data per miRNA for statistical analysis. Missing data were imputed with the k-nearest neighbors imputation approach (k = 5). We wrote the R scripts to run the processing procedures for all datasets. The mRNA expression data were analyzed using affy package from Bioconductor with RMA method. For the gene annotation, genes that correspond to multiple probes were removed and those with unique probe were retained for further analysis.

### Comparison of the outlier detection methods

We compared the performance of five prevailing outlier detection algorithms, COPA (Cancer Outlier Profile Analysis) [21], MOST (Maximum Ordered Subset T-statistics) [22], ORT (Outlier Robust T-statistics) [23], OS (Outlier Sum) [24] and t-test.

All the algorithms were implemented in R scripts written by Lian [22] and Wang [25]. Outliers for each expression dataset were determined by five methods respectively. The threshold for outlier detection was set 0.05 (5%) for all the methods. The outliers detected by at least 3 methods were taken as the putative DE-miRNAs. The proportion of the putative DE-miRNAs in the outlier list found by each method was then calculated. Median value of the percentage among 5 datasets was then calculated as the accuracy for each method.

### Determination of the differentially expressed microRNAs and mRNAs

Outlier microRNAs and mRNAs were detected with MOST, implemented in R scripts by Lian et al. [22]. Outliers that rank top 5% were extracted as differential candidates.

### Target gene prediction for DE-microRNAs

The mRNAs targeted by the DE-microRNAs were obtained from three target prediction algorithms (TargetScan [26], PicTar [27] and miRanda [28]) as well as a database with experimental evidence (miRecord [29]). In order to obtain a more reliable result, we removed the targets found by only one prediction programs.

### Functional enrichment of DE-miRNA targets

We then mapped the target genes of DE-miRNAs to different databases, such as GO (Gene Ontology), KEGG, GeneGo, for functional enrichment analysis. GO and KEGG pathway enrichment were performed using DAVID Bioinformatics Resources 6.7 [30]. GeneGo pathway analysis was performed by MetaCoreTM (GeneGo Inc). In MetaCoreTM, P-values were calculated by hypergeometric distribution to evaluate the statistical significance of the enriched pathways. MetaCoreTM used FDR (False Discovery Rate) adjustment for multiple test correction.

## Results

### Detection of DE-miRNAs with a novel statistical method

T-statistics is most widely used in differential gene expression detection for microarray studies. However, there is a growing realization that the activation patterns of oncogenes are highly heterogeneous. Some oncogenes show altered expressions only in a minor fraction of samples. Tomlins et al. [31] showed that t-statistics has poor detection power for such unconventional circumstance. The problem with t-statistics has motivated a variety of novel analytical methods such as COPA (Cancer Outlier Profile Analysis) [21], MOST (Maximum Ordered Subset T-statistics) [22], ORT (Outlier Robust T-statistics) [23] and OS (Outlier Sum) [24]. In our previous study [32] we have applied these novel methods to prostate cancer microarray datasets. We have demonstrated that the choice of outlier detection method can greatly affect the genes that are identified.

**Figure 2:**
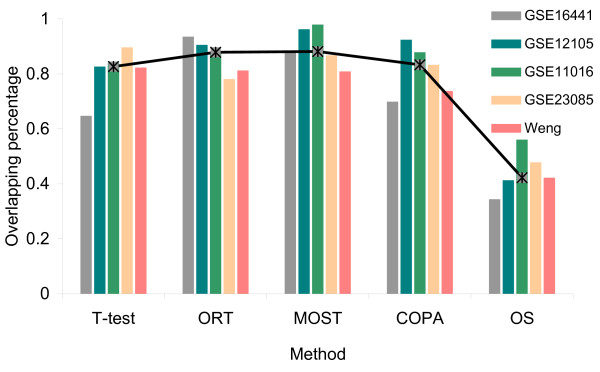
**The percentage of the putative outliers in the original gene list by different methods.** The overlapping percentage was calculated for 5 datasets respectively. The median value among the 5 datasets was defined as the accuracy for the method.

We compared the performance of these methods in outlier detection based on microRNA expression data in ccRCC. Each method was applied to 5 public microRNA profiling datasets (details of these datasets are given in method section) to obtain the outliers. Outliers detected by at least 3 methods were designated as putative DE-miRNAs. The proportion of the putative outliers in the original gene list was then calculated and the median observation among 5 datasets was defined as the accuracy for the method, as illustrated in Figure 2.

Among the five algorithms for comparison, MOST gives best accuracy. ORT also displays favorable performance. COPA performs a little worse than ORT, but still offers some improvement over the t-statistics. The performance of the OS statistics is noticeably worst. The advantage over t-test is apparent for the majority of the novel methods. Thus we come to the conclusion that the newly developed statistics show superior performance in detecting outliers, and therefore provide promising alternatives to the traditional t-statistics. In this study we chose MOST to identify differentially expressed outliers from public datasets. MOST seems to have superior performance when the number of activated samples is unknown [22]. The threshold of outliers is set as 0.05 to select the top 5% of the miRNA outliers.

### Refinement of DE-miRNA lists with the Pipeline of Outlier microRNA Analysis (POMA)

We used an in-house prediction model POMA (Pipeline of Outlier microRNA Analysis) to remove false positive discoveries from the outlier microRNAs detected by MOST. POMA is a model created by our colleagues [33] to evaluate the relevance of microRNAs to given disease conditions. MiRNAs with poor regulatory activity will be excluded from further analysis. POMA is based on two hypotheses: the microRNA activity could be reflected by the deregulated expression of its target genes; if the deregulated genes are targeted exclusively by certain microRNA, that microRNA is more likely to show regulatory activity. The stepwise procedure of POMA is described as follows:

(1) Z_score=α/β

a) We conducted a comprehensive search of all possible microRNA-mRNA interactions for human. Experimentally validated interactions were extracted from 4 databases: miRecords, miRTarbase, miR2Disease and TarBase. Computationally predicted interactions were retrieved from HOCTAR, starBase, and ExprTargetDB. Based on these data, we reconstructed a human microRNA-mRNA interaction network.

b) We reanalyzed 4 public gene expression data in ccRCC vs. normal kidney samples, and identified deregulated genes in ccRCC.

c) The deregulated genes detected in step (b) were subsequently mapped to the microRNA-mRNA interaction network established in step (a), to construct a ccRCC-specific microRNA-mRNA interaction sub-network.

d) We defined a Z_score to measure the probability of microRNA having regulatory role in ccRCC:

α: Number of outlier genes targeted exclusively by a specific microRNA; β: Number of all the outlier genes targeted by that microRNA; (α, β >1).

Z_score is calculated for each candidate microRNA in the sub-network. Using a threshold of 0.1, we identified a list of miRNAs with potential regulatory role in ccRCC for each mRNA expression dataset. The final list was an overlap of the active microRNA identified in at least 3 of the 4 gene expression datasets.

e) The active microRNA list found in step (d) was cross-matched with the DE-microRNA list of each miRNA dataset. The intersected microRNAs (listed in Additional file 2) were retained for further analysis.

### POMA improves inter-dataset consistency

**Figure 3:**
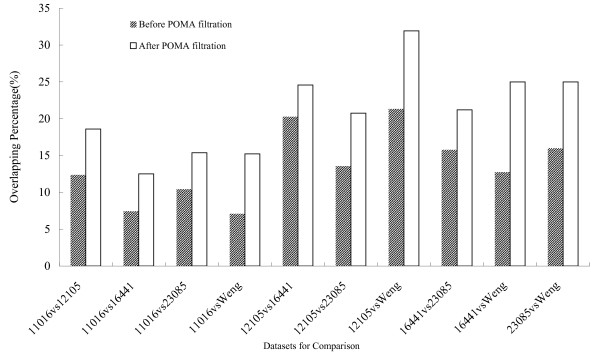
**Pair-wise comparison between 5 datasets at different levels.** X axis shows the 10 pair-wise comparison sets derived from 5 datasets. Y axis denotes the overlapping percentage at different levels.

POMA predicts a list of microRNA that might play regulatory role in ccRCC. These microRNAs serve as a filter that removes the DE-miRNAs without actual function. After filtration, the DE-miRNA list is greatly reduced, resulting in a robust set of functional microRNAs. In addition, a higher overlap between different miRNA datasets was observed. The inter-dataset overlapping percentage before and after POMA filtration is illustrated in Figure 3.

The p-values for the overlapping percentage were 4.38165E-05 by paired t-test, indicating the significance of the difference. Although the number of DE-microRNAs decreased, the consistency between multiple datasets is greatly improved. The enhanced consistency enables us to extract common microRNA expression signatures from different datasets. As a result, we retrieved a set of 11 microRNAs (listed in Table 2), which were shared by at least 4 of the 5 datasets.

**Table 2:** DE-miRNAs with outlier activity in ccRCC pathogenesis

**Human microRNA**	**Accession No.**	**Chr**	**Chromosomal location**	**microRNA cluster**	**Family**	**PubMed citation No.**
hsa-miR-210	MIMAT0000267	11	transcripts	0	210	15
hsa-miR-138-5p	MIMAT0000430	3	intergenic	0	138	5
hsa-miR-16-5p	MIMAT0000069	13	transcripts	1	15	2
hsa-miR-224-5p	MIMAT0000281	X	transcripts	1	224	9
hsa-miR-34a-5p	MIMAT0000255	1	intergenic	0	34	5
hsa-miR-184	MIMAT0000454	15	intergenic	0	184	6
hsa-miR-122-5p	MIMAT0000421	18	intergenic	1	122	6
hsa-miR-126-3p	MIMAT0000445	9	transcript	0	126	5
hsa-miR-155-5p	MIMAT0000646	21	transcript	0	155	14
hsa-miR-15b-5p	MIMAT0000068	3	transcript	1	15	3
hsa-miR-660	MIMAT0003338	X	transcript	6	188	1

Furthermore, we performed a literature search of the 11 microRNA markers to validate their relevance in the regulation of ccRCC. It’s found that all of them have been reported for their roles in renal cell carcinoma. The numbers of supporting literatures for each microRNA are also given in Table 2. These “literature curated” microRNAs provide a focused and robust signature to separate normal from cancerous kidney tissues.

### Targets prediction and functional enrichment

We conducted a high-stringency target prediction for the DE-miRNAs. Target genes were obtained from both experimentally supported databases and prediction algorithms. Number of target genes for each dataset is listed in Table 3. Detailed list of target genes are available in Additional file 3. The targets of each individual dataset were mapped to functional databases, e.g. GO [34], KEGG [35] and GeneGo. Table 3 illustrates the number of various biological themes significantly enriched with target genes for each dataset. Detailed lists of the enriched functional categories and pathways could be found in Additional file 4.

**Table 3:** The number of various enriched biological themes for different datasets

**Dataset**	**DE-miRNA**	**Target**	**GO-BP**	**GO-MF**	**KEGG**	**GeneGo**
			**(FDR < 0.05)**	**(FDR < 0.05)**	**FDR < (0.05)**	**(FDR < 0.001)**
GSE 11016	21	853	18	9	2	99
GSE 12105	22	764	54	11	7	152
GSE 16441	35	1136	110	15	11	149
GSE 23085	31	895	53	11	13	125
Weng	25	921	67	10	9	135
Shared	5	388	8	7	6	62

### Identification of ccRCC related functions and pathways

DE-miRNA target genes are statistically enriched in GO terms of cell cycle and transcription. The top enriched GO terms include: regulation of cyclin-dependent protein kinase activity, transcription, DNA-dependent regulation of transcription, regulation of cell proliferation, sequence-specific DNA binding and transcription regulator activity.

KEGG pathways that are significantly enriched with the DE-miRNA targets were also identified, many of which are associated in cancer, e.g. colorectal cancer, Cell cycle, Neurotrophin signaling pathway, Renal cell carcinoma, Prostate cancer, MAPK signaling pathway, and p53 signaling pathway.

The top GeneGo pathway maps regulated by DE-miRNA converge on cell adhesion, cell cycle and cytoskeleton remodeling, most of which are known to play a part in tumor development. Among the significantly enriched GeneGo pathways (FDR < 0.001), 60 were shared by all of the 5 datasets (see Additional file 4). To evaluate the relevance of these pathway maps in ccRCC, we searched PubMed for the published papers describing their constituent network objects in ccRCC. The network objects with previous literature support were considered to be ccRCC-related.

**Figure 4:**
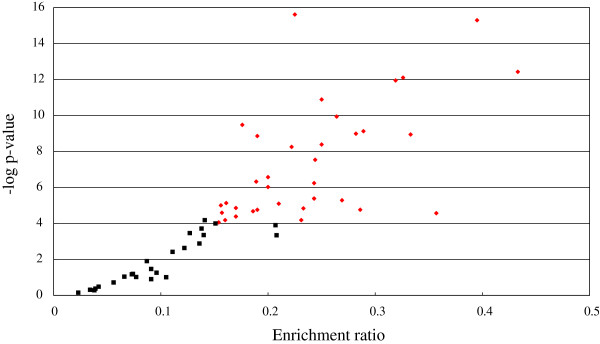
**Volcano plot of pathways enriched with RCC-related genes.** The red points indicate pathways of interest that display both large enrichment ratio (>0.15, x-axis) as well as high statistical significance (P < 0.0001, y-axis).

After text mining, 36 out of the 60 pathways (60%) were found to be highly saturated with well-characterized ccRCC-related objects (enrichment ratio>0.15, p-value<0.0001). To visualize the most significantly enriched pathways we constructed a scatter plot (Figure 4) by plotting the -log10 of p-value versus gene enrichment ratio on the y- and x-axes, respectively. The most meaningful points that display both large enrichment ratio (>0.15, x-axis) as well as high statistical significance (P<0.0001, y-axis) were shown in red. These points could be the potential regulatory pathways in renal carcinogenesis. The top 10 significant GeneGo pathways enriched with ccRCC-related objects are listed in Table 4.

**Table 4:** Top 10 of the significant GeneGo pathways enriched with both DE-miRNA targets and RCC-related genes

**Pathway map**	**Pathway map category**	**Ratio of RCC related objects**	**P-value**	**PubMed citation count**
TGF, WNT and cytoskeletal remodeling	Cytoskeleton remodeling	25/111	2.41E-16	
AKT signaling	Signal transduction	17/43	5.00E-16	70
Brca1 as a transcription regulator	DNA damage	13/30	3.70E-13	
PTEN pathway	Signal transduction	15/46	7.79E-13	22
PIP3 signaling in cardiac myocytes	Development	15/47	1.12E-12	
Regulation of epithelial-to-mesenchymal transition (EMT)	Development	16/64	1.12E-11	12
Influence of Ras and Rho proteins on G1/S Transition	Cell cycle	14/53	1.14E-10	2
Cytoskeleton remodeling	Cytoskeleton remodeling	18/102	3.30E-10	2
Regulation of G1/S transition (part 1)	Cell cycle	11/38	7.43E-10	4
Receptor-mediated HIF regulation	Transcription	11/39	1.02E-9	7

A further PubMed search highlighted 22 out of the 36 putative ccRCC-related pathways with literature support in ccRCC pathogenesis. The remaining 14 pathways without previous annotation are considered to be promising novel pathways contributing to ccRCC (See details in Additional file 5). Among the novel GeneGo pathways, TGF, WNT and cytoskeletal remodeling is most significantly enriched, the pathway map is illustrated in Additional file 6. The pathway is highly saturated with network objects previously found to associate with RCC, such as TCF, AKT, VEGF-A, WNT, Frizzled, TGF-beta, RhoA, Beta-catinin, c-Myc, Cyclin D1 and c-Jun. This pathway focuses on WNT protein family and its downstream effectors. The WNT signaling pathway plays a central role during tumorigenesis and inappropriate activation of this pathway has been observed in many human cancers. Wnt ligands first bind to Frizzled family of Wnt receptors to form Wnt-Frizzled complexes. Upon binding with the Axin-related protein, Wnt regulates the stability of catenin β, which is known to play essential roles at cell-cell adherence junctions. Catenin β then binds to TCF, a family of transcription factors, inducing the transcription of Wnt target genes, such as c-Myc, c-Jun and Cyclin D1. Both c-Myc and c-Jun are oncogenic transcription factors that function in cell cycle progression, apoptosis and cellular transformation. Cyclin D1 is a nuclear protein involved in cell cycle progression in G1/S transition. Activation of these genes contributes to early RCC development. These findings are consistent with the observation that WNT signaling pathway is deregulated during renal carcinoma development. Actin and a variety of actin-binding proteins are also central in the pathway. Remodeling of actins regulates the motility of cells and maintains the cytoskeleton. Cytoskeletal actin disruption is the key factor that triggers apoptosis.

TGF, WNT and cytoskeletal remodeling pathway also contains some objects without previous annotation in ccRCC carcinogenesis, such as ROCK, MEK1, p38, MAPK, axin. These objects could be putative therapeutic targets for novel treatment strategies against ccRCC.

**Figure 5:**
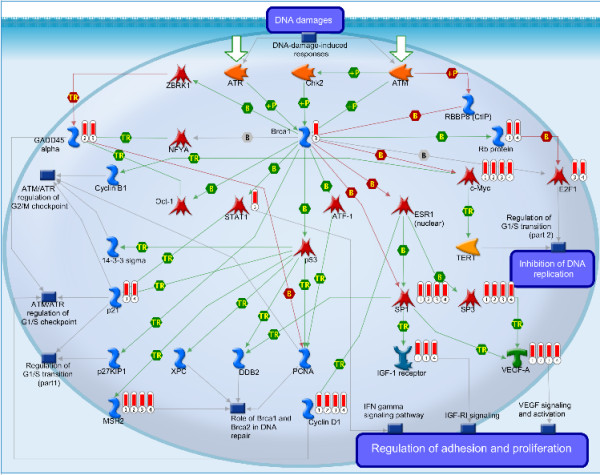
**Graphic illustration of Brca1 as a transcription regulator pathway map.** Red thermometers show an object that can be regulated by a DE-miRNA. The numerical subscript corresponding to the datasets to which the gene belongs. See Additional file 7 for the notation of each sign in this figure.

Another pathway preferentially targeted by the DE-miRNAs is Brca1 as a transcription regulator, which belongs to the DNA damage category. The pathway map is illustrated in Figure 5 (drawn by MetaCore™, see Additional file 7 for the notation of each sign in this figure). It’s evident in Figure 5 that the pathway is enriched with RCC-related objects such as c-Myc, E2F1, Rb protein, IGF1-receptor, VEGF-A and cyclin D1, most of which are predicted targets of miRNAs differentially expressed in ccRCC. The pathway also includes several targets of DE-miRNAs whose association with ccRCC has not been reported before, e.g. Brca1, sp1, sp3, MSH2, p21, GADD45 alpha and stat1. Central in the pathway is the Breast cancer associated gene-1 (Brac1) which plays a central role in DNA repair. BRCA1 is a well-known tumor suppressor gene in breast cancer and ovarian cancers [36], but its role in other cancer types remains elusive [37]. This is the first study demonstrating the tumor suppressor activity of Brac1 in ccRCC. Brca1 regulates the transcription of proteins at DNA repair pathways via transcription factor p53, such as mismatch repair protein MSH2 [38]. Brca1 also participates in cell cycle regulation. In the absence of DNA damage, Brca1 is associated with ZBRK1 in a complex which inhibits transcription of GADD45 alpha. Upon DNA-damage, Brca1 is phosphorylated and dissociated from the Brca1-ZBRK1 repression complex [39]. The released Brca1 stimulates transcription of GADD45 alpha [40]. GADD45 alpha participates in DNA-damage-induced G1/S checkpoint arrest [41] and DNA-damage-induced G2/M checkpoint arrest [42]. In addition, Brca1 regulates the transcription of some other G1/S checkpoint arrest regulators, e.g. p21 and Cyclin D1. Transcription of p21 [43] may be activated through p53 whereas transcription of Cyclin D1 is regulated via c-Myc [44].

### Signatures at pathway level are more consistent

We performed pair-wise comparison between five datasets at different observation levels, including DE-microRNA, target gene, GO-MF (Molecular Function), GO-BP (Biological Process), and GeneGo pathway, respectively. For 5 miRNA expression datasets, 10 pairs are available for comparison. P-values by paired t-test are well below 0.05, indicating that the overlapping percentages at functional level are significantly higher than that at individual DE-microRNA and target gene level.

## Discussion

In this study, we performed systematic and integrative analysis of 5 ccRCC-related microRNA expression datasets, in order to find more reliable expression signatures. We incorporated a novel outlier detection algorithm and a functional microRNA prediction model, into an integrative framework which could enhance the reproducibility of results across multiple datasets.

We first applied a new statistics, MOST, to the identification of differentially expressed microRNAs. It was found that some oncogenes have highly heterogeneous activation patterns and are activated in only a small subset of patient samples. This well explains the inter-dataset inconsistency. These subset-specific cancer genes however, cannot be detected with traditional t-tests. As our previous studies [32,45] have indicated, new statistics generally outperform traditional t-statistics and are therefore more competent for cancer data analysis.

We then used POMA to refine the DE-miRNA list by reducing false discoveries. POMA is designed to find the microRNAs with regulatory activity in ccRCC condition. Those DE-miRNAs without real regulatory activity in the disease were excluded from subsequent analysis. POMA has been employed by our laboratory in the context of prostate cancer (Zhang, unpublished). This model focuses not only on the profile of microRNAs, but also on mRNA transcripts with altered expression in ccRCC. After POMA filtration, final lists of DE-miRNAs are significantly reduced yet improved consistency is observed between the 5 independent datasets.

Finally we obtained a list of 11 DE-microRNAs with regulatory roles. Literature mining confirmed that all of these microRNAs have been reported to be deregulated in renal cell carcinoma, which lends credibility to our list.

We further characterized the concordance of our 11 DE-miRNA list with the results from NGS (Next-Generation Sequencing) technologies. Next-generation sequencing technology facilitates genome-wide miRNA expression profiling at unprecedented speed and accuracy. It also enables discovery of novel miRNAs. After comparison with 3 NGS-based microRNA profiling studies in ccRCC [17-19], we found that our data correlated well with the results of next generation sequencing. For instance, among our 11-miRNA panel, up to 9 miRNAs (82%) were also detected by Osanto et al. [17], except that miR-180 and 660 were not detected. A higher overlap (10 out of 11) was seen comparing with the DE-miRNAs lists by Weng et al. [18], the only mismatch is represented by miR-16-5p. An even better concordance was seen in comparison with the data of Zhou et al. [19]. Here, all of 11 microRNAs were also among the list of deregulated microRNAs.

The comparison with NGS-based data further confirmed that the DE-miRNAs identified by us are authentic cancer related miRNAs in ccRCC and could provide potential biomarkers which await further wet lab validation. Moreover, the general DE-miRNA detection pipeline proposed herein is not limited to ccRCC, but also applicable to a wide range of other diseases.

In order to find novel miRNAs without previous annotation, we also tried to expand the list of POMA filter by lowering the threshold for active miRNA selection. We retrieved active microRNA shared in at least 2 of the 4 mRNA dataset. In this way, a less strict filter with more active miRNA is obtained, which might include some additional novel microRNAs worthy of further investigation. The expanded list of miRNAs is provided as Additional file 8.

The ability of miRNAs to target multiple target genes allows them to induce changes in various pathways and processes, which present a further level of mechanism by which ccRCC may be induced. Overlap analysis at different levels confirms that expression signatures across multiple datasets are more consistent at pathway level than at gene level. It’s been recognized cancer is a highly heterogeneous disease.

Single biomarker is unlikely to dictate diagnosis or prognosis success. Consequently, the future of cancer biomarker might rely on coordinated molecular changes instead [46]. As functionally related genes often display a coordinated expression to accomplish their roles in the cell [47,48], one might expect that the inconsistent microRNA lists, when mapped to higher functional levels, could fall within the same functional modules, pathways or networks [49] and become more consistent.

Functional analysis revealed some biological processes which are preferentially targeted by the DE-miRNAs. Interestingly, the top enriched GO terms are mostly involved in cell cycle regulation (e.g., G1/S transition). Aberrant expression of cell cycle regulators could possibly lead to deregulated cell cycle, which is a hallmark of cancer. It’s showed that cell cycle checkpoint regulators such as cyclins and cyclin-dependent kinases are coregulated by the DE-miRNAs. For example, miR-16 family is reported to trigger a cell cycle arrest by coordinately suppressing multiple cell cycle regulatory genes [50]. It’s worth noting that miR-16 happens to be among the 11 deregulated microRNAs identified in this study. All the evidences above corroborate the validity of the results of the present study.

To evaluate the relevance of the enriched GeneGo pathways in ccRCC, we performed text mining at pathway level as well as object level. Many of the objects that constitute the pathways are known to be critical in the renal carcinogenesis. In addition to the known pathways in RCC tumorigenesis, this study also identified 14 novel ccRCC related pathways. This is the first study demonstrating their relevance in ccRCC. The multiple pathway alterations identified suggest that the miRNAs are potentially regulating many of the necessary steps required by ccRCC development, from changes in the cellular cytoskeleton to regulating cell cycle as well as DNA damage. The cellular functions of these pathways are consistent with the current view on ccRCC pathogenesis. Therefore, these pathways could be a prospective source of novel drug targets and biomarkers. The inhibition of these pathways by synthetic antisense antagomirs provides potential therapeutic interventions in ccRCC.

## Conclusions

In this study we created a bioinformatics framework and applied it to integrative analysis of multiple microRNA expression datasets. The methodology would hopefully improve the reproducibility of miRNAs across independent datasets. The DE-miRNAs and novel pathways identified herein might be candidate biomarkers and drug targets for ccRCC diagnosis and treatment.

## Competing interests

The authors declare they have no competing interest.

## Authors’ contributions

JC carried out the functional enrichment analysis, performed the statistical analysis and drafted the manuscript. DZ participated in the functional enrichment analysis, and draft the manuscript. WZ designed the POMA algorithm. YT, WY and LG participated in the comparison of outlier detection algorithms. BS conceived of the study, and participated in its design and coordination. All authors read and approved the final manuscript.

## Supplementary Material

Additional file 1 Summary of gene expression datasets used in this study. Click here for file

Additional file 2 DE-miRNA list after POMA filtration for each dataset. Click here for file

Additional file 3 Entrez IDs and official symbols of targeting genes for DE-miRNAs of each datasets. Click here for file

Additional file 4 Enriched GO terms, KEGG pathways and GeneGo pathways shared by 5 datasets. Click here for file

Additional file 5 Significant GeneGo pathways enriched with both DE-miRNA targets and curated RCC-related genes. Click here for file

Additional file 6 **Graphic illustration of TGF, WNT and cytoskeletal remodeling pathway map.** Red thermometers indicate an object under regulationof a DE-miRNA. The numerical subscript represents the datasets to which the gene belongs. Click here for file

Additional file 7 **Legend of the symbols in GeneGo pathway map. **This figure provides the notation for each sign in the pathway maps from GeneGo. Click here for file

Additional file 8 Active microRNA shared in at least 2 of the 4 mRNA dataset. Click here for file
