# Supplementary figures and images for: Clear cell renal cell carcinoma associated microRNA expression signatures identified by an integrated bioinformatics analysis
Source: J Transl Med. 2013 Jul 10;11:169. doi: 10.1186/1479-5876-11-169 (PMC3740788; doi:10.1186/1479-5876-11-169)

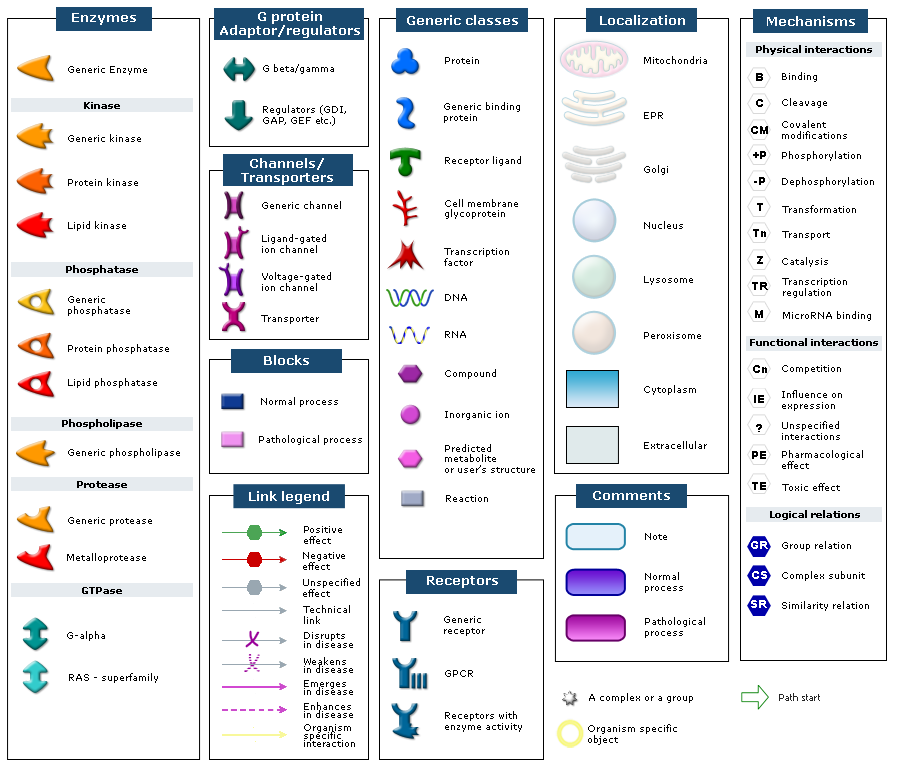

Supplement: Additional file 6 — Graphic illustration of TGF, WNT and cytoskeletal remodeling pathway map. Red thermometers indicate an object under regulationof a DE-miRNA. The numerical subscript represents the datasets to which the gene belongs. [file 1479-5876-11-169-S6.tiff]

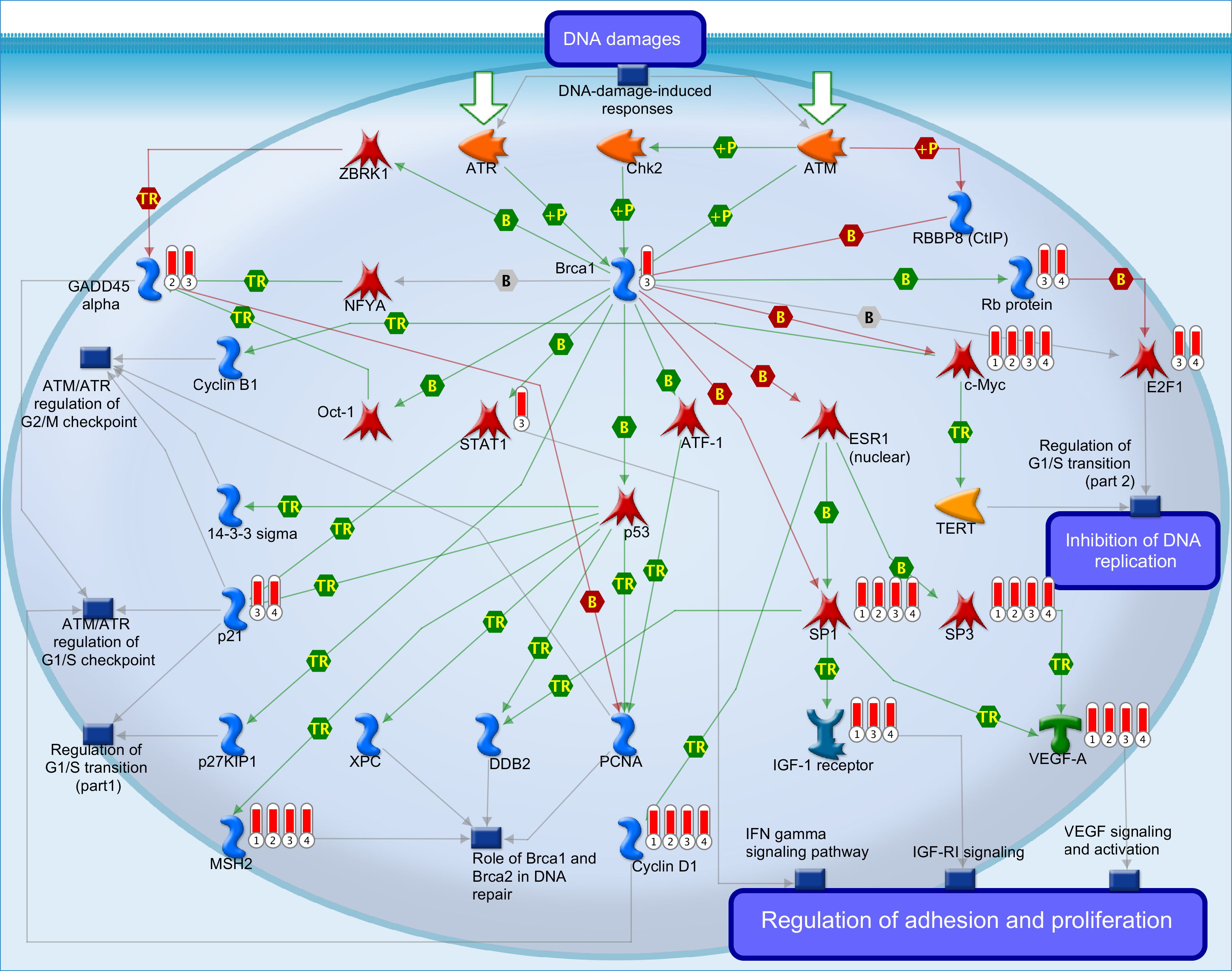

Supplement: Additional file 7 — Legend of the symbols in GeneGo pathway map. This figure provides the notation for each sign in the pathway maps from GeneGo. [file 1479-5876-11-169-S7.png]
